# Optimising our response to a potential H5N1 avian influenza pandemic: preparing to protect people working at the human–animal interface

**DOI:** 10.5694/mja2.52664

**Published:** 2025-04-23

**Authors:** Kerrie Wiley, Catherine King, Holly Seale, Chris Degeling

**Affiliations:** ^1^ University of Sydney Sydney NSW; ^2^ University of New South Wales Sydney NSW; ^3^ Australian Centre for Health Engagement, Evidence and Values University of Wollongong Wollongong NSW

**Keywords:** Occupational health, Prevention and control, Public policy, Vaccination

The Australian federal government announced a $22.1 million investment to increase the availability of pandemic influenza vaccines in the National Medical Stockpile.^1^ This initiative is part of a broader $100 million One Health approach that includes increased agriculture biosecurity response capability, enhanced wild bird surveillance, environmental measures to protect threatened species, and nationally coordinated communications.^2^ Although this is a significant step forward, it is crucial that the preparedness plan extends beyond communications to also fund activities that support the adoption of mitigation measures, including a vaccine.

## Pre‐emptive social science evidence is essential for pandemic preparedness

Cumulative data from 2003–2025 suggest highly pathogenic avian influenza (HPAI) H5N1 has a case fatality rate in humans of around 48%.^3^ Occupational exposure of recent cases to birds and dairy cattle^4^ make occupational groups an immediate priority; however, viral evolution driving increasing mammalian transmission signals future risk to the broader community.^5^ Understanding the drivers and barriers to adoption of preventive behaviours such as mask wearing or vaccination by at‐risk populations is essential, especially during time‐sensitive outbreaks and pandemics. Social science data provide insights into human behaviour which is vital for designing effective interventions and communication strategies that resonate with priority populations. Before the coronavirus disease 2019 (COVID‐19) pandemic, pandemic preparedness focused on frontline health workers, paying less attention to the diversity of frontline workers not involved in health care. Studies focused on the COVID‐19 vaccine uptake and experiences among food systems workers showed stark differences between occupational subgroups working in agriculture, fisheries, meatworks and retail, demonstrating the need for occupation‐specific data to underpin responses tailored to each group's needs.^6^ Studies also identified system inequities resulting in food system workers being underprotected as the COVID‐19 pandemic progressed.^7^, ^8^ Group‐specific knowledge is crucial: the barriers to vaccination and personal protective equipment (PPE) use among poultry workers, for example, may be different to those of wildlife workers such as land and sea rangers. Both groups are at risk for HPAI H5N1 but may have different needs requiring tailored solutions to ensure timely vaccination in the event of an outbreak.

## Social science data are valuable but historically underutilised

The value of social science data in informing public health strategies has long been recognised, as has its historic underutilisation and underfunding.^9^ For example, it was estimated that expenditure on anthropological activity during the 2013–2014 West Africa Ebola response was 0.03% of the total $10 billion,^10^ despite the substantial contribution of social and behavioural insights that led to modifications of cultural norms, such as burial practices, and ultimately a reduction in cases.^11^ Social science was increasingly funded and included in international public health responses to the COVID‐19 pandemic,^12^, ^13^, ^14^ such as Italy's rapid use of national surveys to identify and act on emerging mental health issues resulting from lockdowns.^15^ Funding was withdrawn in most countries during the endemic phase, and mechanisms to collect and use social science data stopped.^14^

## Pre‐emptive social science research should be an explicit part of Australia's avian influenza preparedness investment

Following the COVID‐19 pandemic in Australia, there have been declines in trust in governments and institutions associated with the mandates and restrictions imposed during pandemic responses,^16^ but little is known about how this will affect public willingness to adopt preventive behaviours such as using PPE or accepting vaccination in future. There is some evidence suggesting a decrease in trust on vaccines: a 2022 Australian report found a reduction in the perceived importance of childhood vaccines since the COVID‐19 pandemic;^17^ however, the role of people's experiences with the COVID‐19 vaccine in how a new vaccine would be received is still largely unknown. There is limited specific evidence on what drives or hinders preventive behaviours among groups currently at risk of HPAI working at the human–animal interface, such as veterinarians or agricultural and wildlife workers.^18^ In the context of decreasing trust and vaccine acceptance in the wider community, understanding the acceptability of a new vaccine to these at‐risk groups, especially one using an mRNA platform, is crucial for any pandemic preparedness planning.

## Embedding social science in One Health pandemic preparedness approaches

Globally, One Health is increasingly being embraced as an ideal framework for pandemic prevention, preparedness and response, with attention shifting towards prevention and preparedness following lessons learned from the cost of the COVID‐19 response.^19^, ^20^ Behavioural and social insights should be a key pillar of prevention and preparedness but do not yet occupy a large part of planning. A recent scoping review of One Health adoption in international pandemic prevention, preparedness and response strategies identified that human and animal health were well integrated into the reviewed strategies (> 95% of strategies had these components); however, environmental sciences were less well integrated (58% of strategies) and social sciences were poorly integrated (14% of strategies).^21^

The Australian *COVID‐19 Response Inquiry report* makes several explicit recommendations to enhance the use of social science and communications, including building in‐house social science capacity within the newly established Centre for Disease Control, and establishing structures for evidence synthesis and communication that should be maintained outside of emergencies.^16^ We applaud this and the Australian Government's response in funding environmental measures as part of the aforementioned One Health package. However, the package needs explicit, adequate funding for pre‐emptive social and behavioural data collection for at‐risk groups, in addition to communications strategies to ensure any response is appropriate and acceptable to those groups.

Pathogens are adaptable and unpredictable, and with up to 75% of human pathogens being of zoonotic origin,^22^ people working at the human–animal interface need to be prioritised and the drivers of their current attitudes and behaviours understood to aid rapid and appropriate responses. The recent spread of HPAI H5N1 in North America is a poignant example: as of April 2025, 1009 dairy cow herds and over 167 million birds in commercial and domestic flocks were confirmed infected with H5N1. Seventy human cases, including one death, were confirmed, with 41 dairy workers and 24 poultry workers all occupationally exposed to infected livestock.^23^ Studies show that the use of recommended PPE was low among dairy workers in the weeks before and after virus detection, and workers reported using alternative items such as sunglasses and bandanas.^24^

Even with high levels of biosecurity, the increasing intensification of agricultural systems will heighten zoonotic risks in the future.^25^ Investing in pre‐emptive social research is necessary for effective pandemic planning and responses. Developing knowledge and awareness about vaccines, new technologies and PPE for and with people who are most likely to need them in the future is critical. By funding pre‐emptive social research, the Australian Government can ensure sound pandemic preparedness strategies to protect at‐risk individuals working at the human–animal interface, their close contacts, and food security while building trust in public health interventions, ultimately leading to better health outcomes for all Australians.

## Open access

Open access publishing facilitated by The University of Sydney, as part of the Wiley – the University of Sydney agreement via the Council of Australian University Librarians.

## Competing interests

Holly Seale has previously received funding for investigator‐driven research from Moderna, as well as funding to attend meetings and conferences from Pfizer and Moderna.

## Provenance

Not commissioned; externally peer reviewed.
